# Enhanced Multiferroic Properties of YMnO_3_ Ceramics Fabricated by Spark Plasma Sintering Along with Low-Temperature Solid-State Reaction

**DOI:** 10.3390/ma10050474

**Published:** 2017-04-28

**Authors:** Meng Wang, Ting Wang, Shenhua Song, Muchakayala Ravi, Renchen Liu, Shishan Ji

**Affiliations:** 1 Shenzhen Key Laboratory of Advanced Materials, Department of Materials Science and Engineering, Shenzhen Graduate School, Harbin Institute of Technology, Shenzhen 518055, China; wangmeng1985@hit.edu.cn (M.W.); twang-hawk@foxmail.com (T.W.); raviphd@yahoo.com (M.R.); 2 Research Institute of Tsinghua University in Shenzhen, Shenzhen 518055, China; renchen.liu@gmail.com; 3 Tsinghua Innovation Center in Dongguan, Dongguan 523808, China

**Keywords:** multiferroic materials, spark plasma sintering, low-temperature solid-state reaction, dielectric properties, ferroelectric properties, magnetic properties

## Abstract

Based on precursor powders with a size of 200–300 nm prepared by the low-temperature solid-state reaction method, phase-pure YMnO_3_ ceramics are fabricated using spark plasma sintering (SPS). X-ray diffraction (XRD) and scanning electron microscopy (SEM) reveal that the high-purity YMnO_3_ ceramics can be prepared by SPS at 1000 °C for 5 minutes with annealing at 800 °C for 2 h. The relative density of the sample is as high as 97%, which is much higher than those of the samples sintered by other methods. The present dielectric and magnetic properties are much better than those of the samples fabricated by conventional methods and SPS with ball-milling precursors, and the ferroelectric loops at room temperature can be detected. These findings indicate that the YMnO_3_ ceramics prepared by the low temperature solid reaction method and SPS possess excellent dielectric lossy ferroelectric properties at room temperature, and magnetic properties at low temperature (10 K), making them suitable for potential multiferroic applications.

## 1. Introduction

Multiferroic material, which possesses ferroelasticity, ferroelectricity, and ferromagnetism, has become one of the most important research interests in functional ceramics. In particular, it possesses the magnetoelectric effect, and is deemed as the next generation of electronic and magnetic devices [1,2]. Among these, YMnO_3_ is one of the most advanced multiferroic materials; it belongs to the P6_3cm_ space group with a relatively high Curie temperature (T_C_ ~ 900 K) and a low Neel temperature (T_N_ ~ 70 K). Moreover, it is proved that YMnO_3_ can couple both ferroelectric and antiferromagnetic properties [3,4,5,6,7], with potential applications in telecommunication and data storage.

However, fabrication methods restrain the preparation of high-quality YMnO_3_. Most studies adopted the conventional solid-state method to fabricate YMnO_3_. The main drawbacks of this method are: (1) the preparation process includes complex procedures, such as preheating, ball-milling, calcination, sintering, and heat treatment; (2) high sintering temperature and long sintering time are necessary. Usually, a high temperature of 1300–1400 °C and a sintering time of more than 10 h are required to prepare high-density YMnO_3_ ceramics; and (3) some secondary phases are prone to be generated during the fabrication process. In the range of 800–1200 °C, the secondary phases, such as Y_2_O_3_, MnO, and Y_2_Mn_5_O_12_ always form during the sintering process. The bottlenecks of the traditional solid-state sintering method limit the potential application of YMnO_3_ ceramics.

Another common precursor fabrication method is the sol-gel method. In this method, the homogeneity of the reagents can be acquired at a molecular or atomic level, and the reaction temperature can be lowered. The fabricated particles can reach nano scale and the surface activation energy is high, so sintering temperature can be decreased and the grain size of ceramics can be reduced. Han et al. [8], Ahmad et al. [9], and Zhang et al. [7] successfully prepared YMnO_3_ powders by this method. The main drawbacks of this method are: (1) the processing time is still long, which can last several hours or even days; (2) when the precursor powders prepared by this method are used to fabricate ceramics, more defects are generated during the process of gas generation and expelling, resulting in a material with a relatively low density; (3) the as-synthesized compounds are easily decompose during the sintering period; and (4) the preparation cost is high and the pollution is severe [3,10,11]. So a simple and high efficiency method should be used for YMnO_3_ precursor fabrication.

Recently, spark plasma sintering (SPS) has been extensively adopted as an advanced route for material fabrication. SPS is now referred to as pulsed electric current sintering (PECS). The principle of this technique is that micro-sized electric charges are generated between particles under uniaxial pressure, so a densified structure can be obtained with uniaxial pressure to consolidate powders. This technology was used to get cleaner grain boundaries in sintered ceramic materials [12,13], higher permittivity in ferroelectrics [14], excellent magnetic properties [15], higher thermoelectric properties [16], and reduced impurity segregation at grain boundaries [12]. As for multiferroic ceramics fabrication, SPS can allow a low sintering temperature (200–300 K lower than that of the conventional method) and short sintering time [17,18,19,20]. Up to now, only Ma has studied YMnO_3_ ceramics sintered by SPS [10].

In view of this situation, a method for precursor fabrication, namely the low-temperature solid state reaction method, has the priority of low processing temperature, pure phase production, low cost, easy fabrication procedure, and ultrafine particle size. It only needs grinding in an agate mortar to fabricate precursors, so the procedure is greatly simplified. This method was successfully adopted to obtain YMnO_3_ precursors [21]. However, low-temperature solid-state reaction methods combined with SPS was not adopted to fabricate YMnO_3_ pallets. In this work, an SPS technique combined with the low-temperature solid-state reaction precursor was applied to prepare YMnO_3_ ceramics. The microstructures of the YMnO_3_ precursor and ceramics were studied extensively. The dielectric, ferroelectric, and magnetic properties were also investigated.

## 2. Experimental Procedures

YMnO_3_ powders were synthesized via the low-temperature solid reaction method; the raw reagents include Mn(CH_3_COO)_2_∙4H_2_O, Y(NO_3_)_3_∙6H_2_O, and citric acid. Initially, Mn(CH_3_COO)_2_∙4H_2_O, Y(NO_3_)_3_∙6H_2_O, and citric acid under a mole ratio of 1:1:2 were weighed and ground in an agate mortar for half an hour, respectively. Then the respective powders were mixed and ground again in an agate mortar for half an hour. A light brown viscous substance was formed during the grinding process. The viscous substance was heated at 120 °C for 2 h and a powdery material was harvested as the precursor material. The powders were ground and subsequently calcined for 1 h in air at 800 °C to provide what shall be referred to as the pure phase YMnO_3_ powders, serving as raw precursors for the subsequent SPS process. In the SPS process, the YMnO_3_ powders were placed in a graphite die and heated at a rate of 100 °C/min from room temperature to 1000 °C, followed by sintering for 5 min under an atmospheric pressure of 10^−2^ Pa. During the entire SPS process, a uniaxial pressure of 50 MPa was constantly maintained on the sample [22]. After completing the sintering process, pellet-shaped samples were formed. The samples were then polished to a size of 2 mm thick and 10 mm diameter, followed by annealing at 800 °C for 2 h in air in order to recover the oxygen stoichiometric ratio, release strain, and remove carbon contamination.

The crystal structures of the resulting samples were characterized by X-ray diffraction (XRD) using a Rigaku diffractometer with Cu-K radiation (D/max-RB, Rigaku, Tokyo, Japan). The densities of the samples were determined by the Archimedes method using distilled water as the immersion liquid. Both the microstructure of the powders and the fracture surface of the pellets were examined using field emission scanning electron microscopy (S-4700, Hitachi, Tokyo, Japan). For dielectric measurements, all the samples were polished and coated with silver paint. To improve the paint conductivity and make the contact between the paint and sample better, the paint was cured for 2 h at 120 °C. The dielectric properties of the samples were measured using a computer-controlled impedance analyzer (PSM1735, Newton 4th Ltd., Newton, UK). The ferroelectric properties of the samples were measured using a ferroelectric analyzer (Premier-II, Radiant Technologies, Inc., Albuquerque, NM, USA). Magnetic properties of the samples were obtained using a physical property measurement system (DynaCool-9T, Quantum Design, Leatherhead, Surrey, UK) at low temperature (10 K).

## 3. Results and Discussion

**Figure 1:**
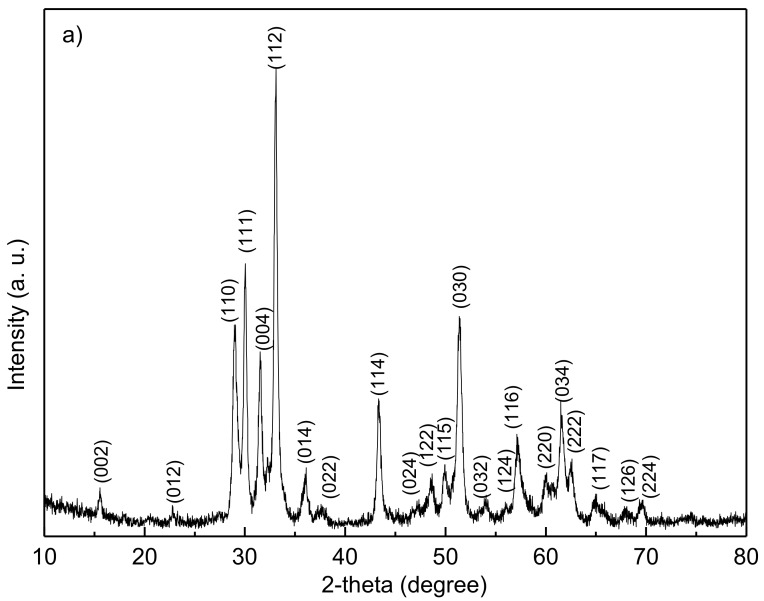

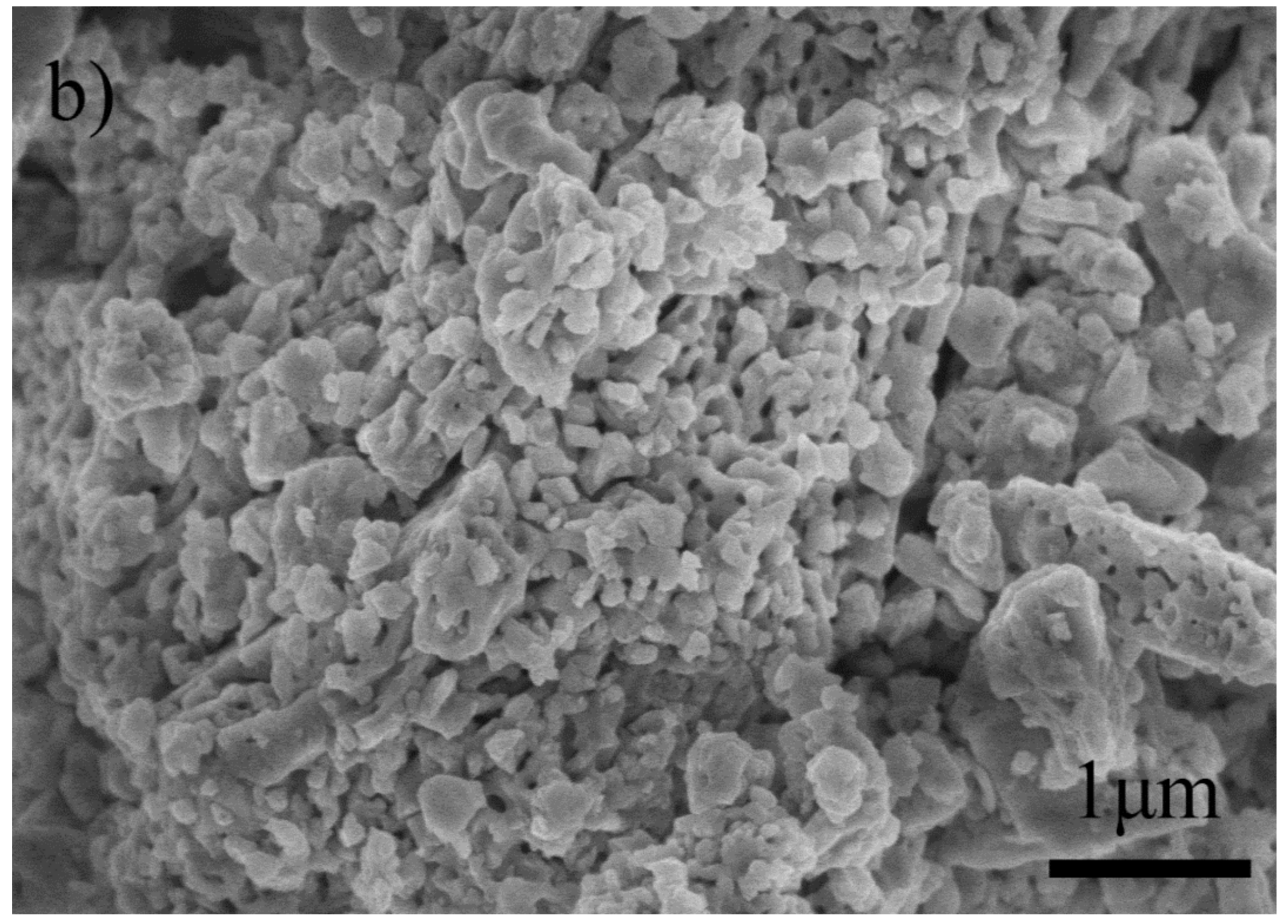
(**a**) XRD pattern and (**b**) SEM micrograph for YMnO_3_ powders.

Figure 1a shows the XRD pattern of the precursor powders synthesized by the low-temperature solid-state reaction method. All the diffraction peaks are well indexed with the YMnO_3_ phase and possess a hexagonal structure. No impure phases (such as Y_2_O_3_, Mn_2_O_3_, or MnO) were revealed in the powders within the limits of the XRD machine capability. Figure 1b shows the SEM micrograph of the YMnO_3_ precursor powders. Although some agglomeration was present, the particle size was homogeneous and about 200–300 nm. Therefore, submicron-size precursor powders were obtained.

**Figure 2:**
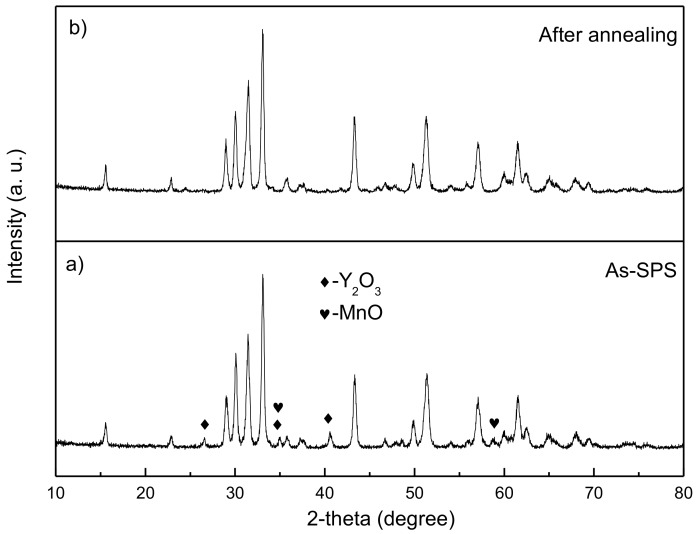
XRD patterns for YMnO_3_ samples (**a**) as SPS and (**b**) after annealing.

XRD patterns of the SPS-prepared and annealed sample are shown in Figure 2a,b, respectively. According to the XRD index, the major phase is the hexagonal YMnO_3_, and there is a minor amount of secondary phase of MnO (see Figure 2a). It is common because YMnO_3_→Y_2_O_3_ + Mn_2_O_3_ can always occur in the range of 800–1200 °C, and Mn_2_O_3_ is easy to transform to MnO with the environment of oxygen deprivation under the vacuum atmosphere of SPS [6,7,8,10,23]. Therefore, to obtain pure YMnO_3_, a higher or a lower annealing temperature must be selected. As shown in Figure 2b, YMnO_3_ ceramics without any impurities are synthesized after annealing at 800 °C for 2 h in air. Compared with the traditional methods, the present method requires a much lower sintering and annealing temperature to obtain a pure phase, so the preparation procedure is greatly simplified, and it is an efficient way to save energy.

**Figure 3:**
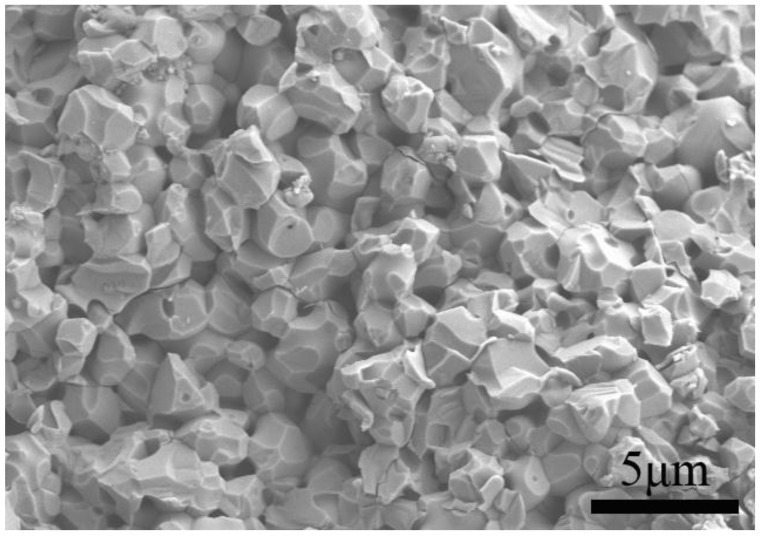
SEM micrograph of fracture surface of the YMnO_3_ ceramics.

A SEM micrograph of the fracture surface of the sample is represented in Figure 3. The sample exhibits a highly dense structure with smooth facets, proving that the ceramics are completely sintered. After the SPS and annealing process, the grain size of the sintered ceramics reaches ~1–2 μm and few defects and cracks can be observed. Compared with YMnO_3_ ceramics with a grain size of 3–5 μm prepared by SPS with ball-milling precursors [10], the ceramics in the present study possess more refined grains.

The density of the YMnO_3_ pellets was also studied. Before annealing, the density of the sample reaches a reasonably high value of 4.910 g/cm^3^, which is 95.7% of the theoretical value (theoretical density = 5.126 g/cm^3^). After annealing for 2 h in air, the sample exhibits a slightly higher density (4.972 cm^3^, 97.9% of the theoretical value) than that before annealing. This is because the remaining minor impurities can continue to transform to YMnO_3_, and the carbon contamination is diminished during the annealing treatment. The present density is higher than that of YMnO_3_ ceramics fabricated by the traditional solid-state sintering [24] and that of YMnO_3_ ceramics prepared by SPS with ball-milling precursors [10].

**Figure 4:**
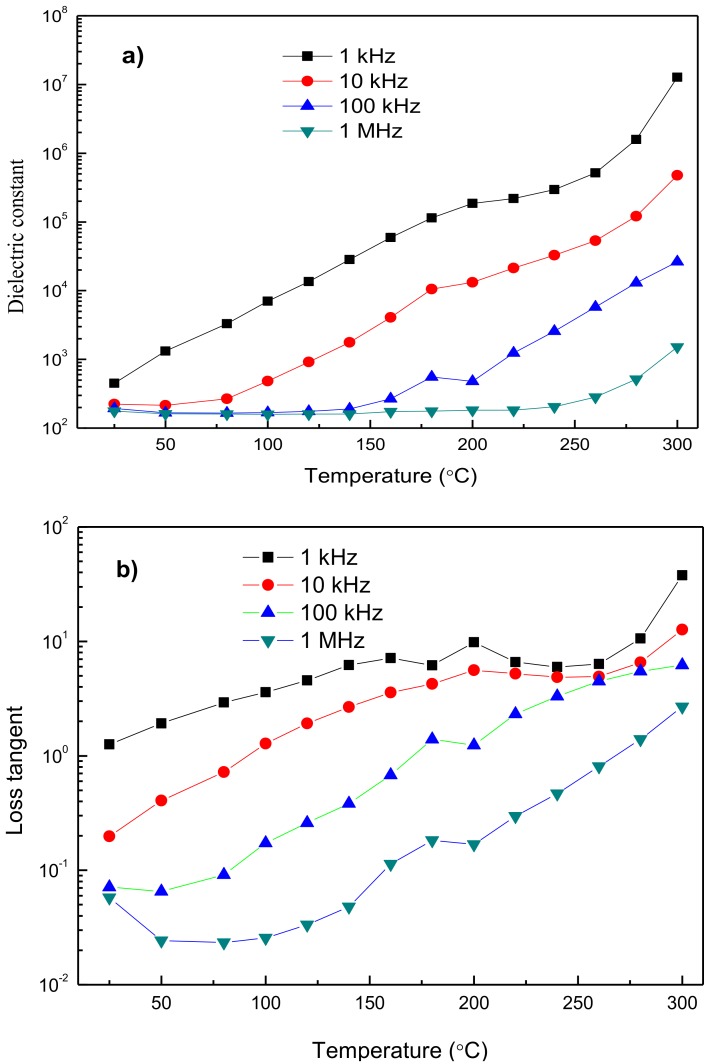
Temperature dependence of (**a**) dielectric constant and (**b**) dielectric loss under frequencies of 1, 10, 100, and 1000 kHz for the YMnO_3_ ceramics.

Figure 4 shows the temperature dependences of the dielectric constant and loss at the frequencies of 1 kHz, 10 kHz, 100 kHz, and 1 MHz; it shows that both the parameters increase with increasing temperature. At the same temperature, both parameters decrease with the increase of frequency. In addition, the dielectric constant remains almost constant until 160 °C and starts to increase sharply with further increasing temperature. An obvious dielectric relaxation effect can be observed in the range of test temperature, and it is postponed to higher temperatures as the frequency increases. In addition, a small peak of dielectric loss can be viewed around 160–200 °C. This peak is ascribed to hopping charge, which is also reported by Ma et al. [10] and Zhang et al. [7]. Usually, the hopping process is related to the valance variation of Mn ions and oxygen vacancies, thereby resulting in the dielectric relaxation phenomenon. But compared with other reports, the dielectric constant is obviously higher, and the dielectric loss is lower in the present study, proving that the YMnO_3_ ceramics with high density, few defects, and low impurities were prepared in this study. Generally, the dielectric constant of the present study was much higher than those of YMnO_3_ ceramics prepared by SPS with ball-milling precursors [10]. The reasons for the higher dielectric constant in our study could be: (1) the densities are much higher; and (2) few defects, especially pores, emerge [7,25].

**Figure 5:**
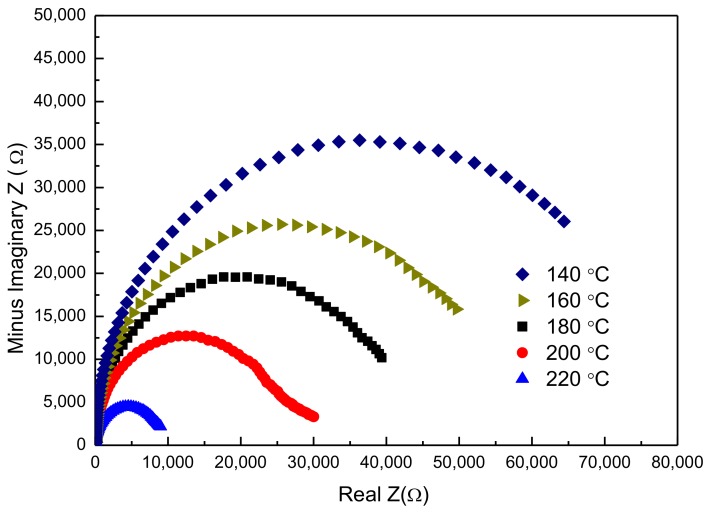
Complex impedance plots at different temperatures.

**Figure 6:**
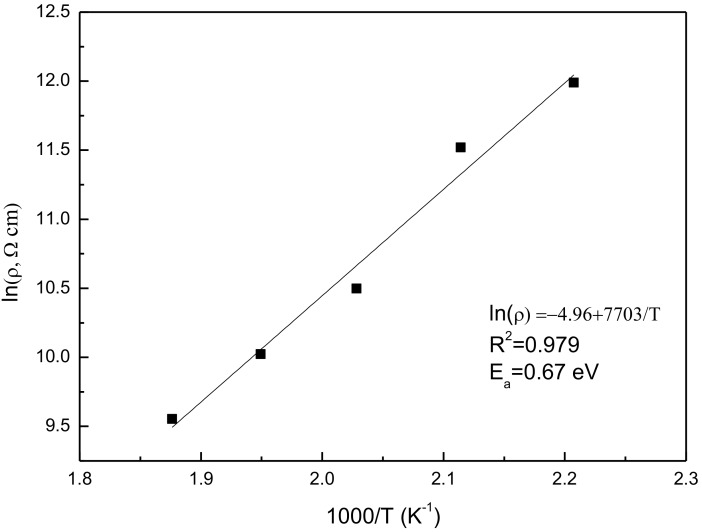
Temperature dependence of resistivity for YMnO_3_ ceramics.

In order to prove the quality of the SPS-prepared samples with annealing, complex impedance plots at several temperatures are represented in Figure 5. Obviously, with increasing temperature, the spectra can exhibit a single and almost ideal semicircular arc whose high-value intercept on the real axis determines the resistance of the sample. Based on the resistance obtained from the complex impedance plots, the resistivity (ρ) is calculated. ln (resistivity) (ln (ρ)) is plotted as a function of reciprocal temperature (1/T) in Figure 6. Clearly, there is a linear relationship between ln (ρ) and 1/T, meaning that the temperature dependence of resistance follows an Arrhenius equation. The equation for the sample is given by

(1) lnρ=−4.96+7703/T

where T is the absolute temperature. With the assumption that the obtained Arrhenius equation is valid at room temperature (298 K), the resistivity is 1.1 × 10^9^ Ω cm. This is much higher than those of other reports. The high resistivity can guarantee better dielectric and ferroelectric properties.

**Figure 7:**
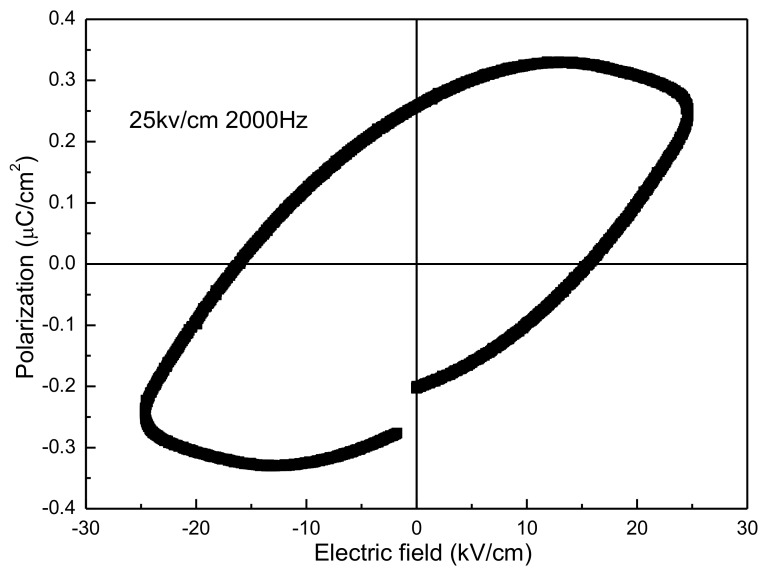
Ferroelectric hysteresis loop of the YMnO_3_ ceramic at room temperature.

The ferroelectric hysteresis loop of the sample at room temperature is shown in Figure 7. The loop has in an ellipse shape, revealing that the sample shows ferroelectricity with a relatively high leakage current. As [26], this kind of loop can only be named a lossy hysteresis loop rather than a saturated ferroelectric hysteresis loop. Normally, a fully saturated ferroelectric hysteresis loop is not able to be viewed for YMnO_3_ at room temperature because of a high leakage current induced by defects and secondary phases [10]. The desired resistivity is 10^10^ to 10^11^ Ω cm and the value in the present study is about 10^9^ Ω cm, so the hysteresis loop is not fully saturated. Up until now, no saturated ferroelectric hysteresis loops at room temperature for the YMnO_3_ samples prepared by conventional and SPS methods were reported. The loops in this study are comparable with ones described in the literature which were tested at around 150–200 K [4,10]. The comparably better ferroelectric properties of the samples in the present study are owing to the low concentration of defects and impurities, and the high density. If a better and saturated ferroelectric loop is obtained, doping will be a good choice.

**Figure 8:**
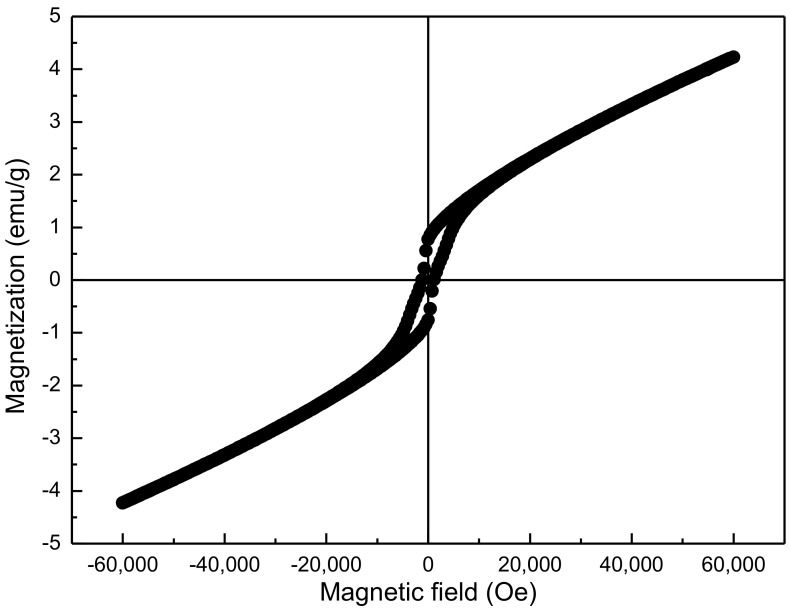
Magnetic hysteresis loop of the YMnO_3_ ceramic at 10 K.

The magnetic hysteresis loop of the YMnO_3_ ceramic at low temperature (10 K) is shown in Figure 8. It is well known that ionic structure determines the antiferromagnetic properties of YMnO_3_. In this structure, one Mn^3+^ ion is surrounded by O^2+^, forming a bipyramid. The Y^3+^ ions are surrounded by eight O^2+^ (only two apical oxygen atoms). The bipyramids are not exactly parallel to the c-axis, thus, the two Y-O apical bond lengths are not the same because of this tilt [27]. The Mn^3+^ magnetic moment is not parallel to the moments of the nearest Mn^3+^ neighboring moments, thus, they form a small angle. This leads to the generation of weak ferromagnetic characteristics in antiferromagnetic YMnO_3_ ceramics [23]. The maximum magnetization (*M*_m_), remnant magnetization (*M*_r_), and coercive field (*H*_c_) are about 4.19 emu/g, 0.77 emu/g, and 1000 Oe, respectively. Compared with the results of other literature (see Table 1), the values of *M*_r_ and *M*_m_ are much higher and *H*_c_ is the same order of magnitude because of the smaller grain size and fewer impurities.

## 4. Conclusions 

Multiferroic YMnO_3_ ceramics are successfully fabricated by a combination of SPS and a low-temperature solid state reaction precursor. Compared with the conventional solid state method and SPS combined with the ball milling precursor method, the synthesis process is greatly simplified, and high-density YMnO_3_ ceramics with fine grains are obtained within a short time and at low temperature. The prepared YMnO_3_ ceramic possesses a high dielectric constant and low dielectric loss, and the resistivity is about 10^9^ Ω cm. The ferroelectric hysteresis loop at room temperature is lossy. The maximum and remnant magnetization at 10 K are about 4.19 emu/g and 0.77 emu/g. All the properties are comparable with results from other literature. The present YMnO_3_ ceramics indicate good dielectric, ferroelectric, and ferromagnetic characteristics, making them suitable for potential multiferroic applications.

## Figures and Tables

**Table 1:** Comparison of the relevant magnetic parameters for YMnO_3_.

No.	*M*_m_ (emu/g)	*M*_r_ (emu/g)	*H*_c_ (kOe)	Test Condition	Reference
1	2.5@7T	0.05	4.1	10K	[28]
2	0.75@3T	0.04	0.98	5K	[7]
3	1.01@2T	0.30	1.83	10K	[10]
4	4.19@6T	0.77	1	10K	Present work
